# Dietary intake among youth adhering to vegan, lacto-ovo-vegetarian, pescatarian or omnivorous diets in Sweden

**DOI:** 10.3389/fnut.2025.1528252

**Published:** 2025-03-10

**Authors:** Isabelle Mulkerrins, Anine Christine Medin, Synne Groufh-Jacobsen, Claire Margerison, Christel Larsson

**Affiliations:** ^1^Department of Food and Nutrition, and Sport Science, Faculty of Education, University of Gothenburg, Gothenburg, Sweden; ^2^Department of Nutrition and Public Health, Faculty of Health and Sport Sciences, University of Agder, Kristiansand, Norway; ^3^Institute for Physical Activity and Nutrition (IPAN), Deakin University, Geelong, VIC, Australia

**Keywords:** plant-based, macronutrient intake, food group intake, food-based dietary guideline, youth

## Abstract

**Objective:**

To assess the intakes of food groups, energy, and macronutrients among youth in Sweden who adhere to vegan, lacto-ovo-vegetarian, pescatarian or omnivorous diets. Further, to evaluate youth’s adherence to the food-based dietary guidelines (FBDG).

**Design:**

In this cross-sectional study, dietary intake data was obtained through repeated non-consecutive 24-h dietary recalls (24HDR) and a dietary screener assessing consumption frequency of food groups. Usual daily intakes were estimated using the Multiple Source Method (MSM), and for usual intakes of food groups the 24HDR intake data was combined with consumption frequency.

**Setting:**

Gothenburg, Sweden, December 2022–January 2024.

**Participants:**

In total 235 youth (78% female, mean age 22 ± 2 years), consisting of 60 vegans, 59 lacto-ovo-vegetarians, 55 pescatarians, and 61 omnivores.

**Results:**

For usual intakes (median value), both g/d and g/MJ, all plant-based dietary groups had higher intakes of legumes and plant-based meat analogs compared to omnivores (for all, *p* < 0.001), and vegans and lacto-ovo-vegetarians had higher intakes of plant-based dairy substitutes (vs. pescatarians and omnivores, *p* < 0.001). Moreover, vegans had higher intakes of refined grain products (vs. pescatarians, *p* = 0.012), nuts/seeds (vs. pescatarians and omnivores, *p* = 0.002), and vegetable oil (vs. omnivores, *p* = 0.014). Omnivores had higher intakes of fried/premade potato dishes (vs. lacto-ovo-vegetarians and vegans, *p* < 0.001), and lower intakes of plain potatoes (vs. lacto-ovo-vegetarians and pescatarians, *p* < 0.001). Overall intakes of ‘sweets and snack foods’ did not differ between dietary groups. Omnivores had higher usual intakes of energy compared to lacto-ovo-vegetarians and pescatarians (10 vs. 9 MJ/d, *p* = 0.016). Most macronutrient recommendations were met across groups, except for carbohydrates (below for omnivores), fiber (below for omnivores and pescatarians), and saturated fatty acids (exceeded by all except vegans). For the FBDG for whole grains, omnivores (23%) had a higher adherence vs. vegans (2%) and lacto-ovo-vegetarians (3%), *p* < 0.001. No difference was found between dietary groups for adherence to the FBDG’s for fruits, berries, and vegetables (10%), nuts (24%), and vegetable oil (4%).

**Conclusion:**

Swedish youth, regardless of dietary practice, need to increase intakes of fruits, berries, vegetables, nuts, and whole grains, and limit consumption of discretionary foods to better align with food and nutrition recommendations.

## 1 Introduction

Unhealthy dietary habits, characterized by high quantities of red and processed meat and limited plant-based foods (i.e., fruits, vegetables, and whole grains), are one of the top modifiable risk factors contributing to poor health and the global burden of disease (1). Simultaneously, these habits are major contributors to environmental destruction (2, 3). Evidence demonstrates that a global transition toward a diet mostly or entirely composed of plant-based foods is part of the solution to reduce both diet-related non-communicable disease (2, 4, 5) and negative environmental impact from dietary intake (2, 3, 6). With regards to this, for long-term health, the Nordic Nutrition Recommendations 2023 (NNR2023) recommend intakes of ≥500 g/d fruits, berries, and vegetables (F&V), ≥90 g/d whole grains, 20–30 g/d nuts, 25 g/d vegetable oil, ≥350 g/d low-fat milk and dairy products, and 300–450 g/week of fish, including 200 g/week from oily fish as well as limited intake of free sugars, salt, and alcohol (7). NNR2023 also state that legumes should constitute a significant part of the diet and intakes of red meat should be below 350 g/week, whereof processed meat should be limited (7).

In recent years, the interest in plant-based diets such as vegan, lacto-ovo-vegetarian, pescatarian and flexitarian has increased in westernized countries (8). In Nordic countries, an estimated 3–8% of young people, based on national dietary surveys, adhere to lacto-ovo-vegetarian or vegan diets in Sweden (17–18 years) (9), Finland (16–18 years) (10), and Norway (18–29 years) (11). As youth (12) are in a stage of life with increased autonomy over their own dietary choices, and most often choose to adopt plant-based diets for reasons other than health (13, 14) including ethical, environmental, financial, or food preferences, the dietary intake is likely to be heterogenous, which could potentially influence diet quality. The overall evidence among adults and children/adolescents indicates that plant-based diets can provide nutritional benefits such as higher dietary fiber and lower saturated fatty acids (SFA) but may also increase the risk of inadequate intakes of some key micronutrients, whereof vitamin B_12_, vitamin D, calcium, iron, zinc, iodine, and selenium (15–18). However, these nutritional outcomes depend on the types and quantities of foods consumed, supplement use, and bioavailability in foods.

Studies on the dietary intake among youth eating plant-based compared to omnivorous diets are limited, and in Sweden it was last assessed in the late 1990’s (19). Given the increased supply of convenient and nutritionally diverse plant-based foods (20–23) up-dated knowledge is required on the dietary intake among current youth eating plant-based diets. Therefore, our objective was to assess the intakes of food groups, energy, and macronutrients among youth in Sweden who adhere to vegan, lacto-ovo-vegetarian, pescatarian or omnivorous diets. Further, to evaluate the adherence to the FBDG’s by NNR2023 among youth with different dietary practices.

## 2 Materials and methods

### 2.1 Recruitment and study eligibility

Between December 2022 and January 2024, healthy 16 to 24 year olds living in Gothenburg, Sweden, or nearby municipalities were recruited by convenience and snowball sampling. Various recruitment methods were employed. Posters about the study were placed in high-schools, universities, libraries, cafes, training centers, gyms, and outside poster boards. Information about the study was shared via newsletters, e-mail lists, and social media platforms. Paid advertisements were utilized on Instagram and Facebook, targeting 18–24 year olds. Physical recruitment occurred at high schools, a public science festival, as well as sports and sustainability events for students.

To be eligible for participation, the youth had to ‘be between the age of 16–24 years’, ‘be healthy with no chronic or acute disease’, ‘have adhered to their current dietary practice (vegan, lacto-ovo-vegetarian, pescatarian, or omnivore) for a minimum of 6 months and have no intention to alter their current dietary practice’, ‘not be pregnant, lactating, or have children’, ‘comprehend Swedish’, and ‘agree to physically visit the research facility in Gothenburg for participation’.

### 2.2 Sample size

*A priori*, sample size was calculated (24) for the primary outcome, energy intake (EI), using data from the Swedish national food consumption survey among youths aged 17–18 years, across sexes (25). To detect a difference of 2.1 MJ between groups with a power of 80%, 42 people are needed in each dietary group, and therefore to account for dropouts we aimed to recruit 60 youth per dietary group.

### 2.3 Study design

All participants visited the research facility at the University of Gothenburg once to partake in the research project named *VeggiSkills-Sweden,* which used a cross-sectional mixed-methods design. During the visit, anthropometrics were measured, blood and urine samples were collected, and an interview-administered 24-h dietary recall (24HDR) was completed. In addition, during the visit participants were asked to fill in a 255-item web-based questionnaire which assessed dietary habits in the past 6 months [i.e., dietary practice, animal-sourced foods included in the diet, mealtime frequency, supplement use, and consumption frequency of food groups using a revised dietary screener (26)], food literacy competencies (general nutrition knowledge, critical nutrition literacy, food skills), food choice motives, health and lifestyle habits (i.e., tobacco use, frequency of physical activity) and sociodemographic information (e.g., parental education level). In this paper, data about dietary intake, anthropometrics, health and lifestyle habits, and sociodemographic information are presented.

### 2.4 Categorization into dietary groups and exclusion of participants

Participants were asked to self-identify their current dietary practice in the web-based questionnaire. To categorize participants into dietary groups their self-identified dietary practice was cross-checked with their responses to a question in the web-based questionnaire which assessed animal-sourced foods included in their diet in the previous 6 months. Participants filled in the questions, “How often have you included ‘milk and/or dairy products’, ‘eggs and/or foods containing eggs’, ‘fish, seafood, and/or fish products’, ‘poultry and/or poultry products’, ‘red meat and/or red meat products’, in your diet in the past six months?.” If they self-identified a vegan dietary practice and selected ‘never’ to all the options, they were categorized into the vegan dietary group (27). If they self-identified an ‘ovo-vegetarian’, ‘lacto-vegetarian’, or ‘lacto-ovo-vegetarian’ dietary practice and reported consumption of milk and/or dairy products and/or eggs, but no fish/seafood or meat (all types) they were categorized into the lacto-ovo-vegetarian dietary group (27). If they self-identified a pescatarian dietary practice and reported consuming fish, seafood and/or fish products, but no intake of meat (all types) they were categorized into the pescatarian dietary group, regardless of intake of eggs and milk/dairy products (27). If they self-identified an omnivorous dietary practice and reported consuming any type of meat and other animal-sourced foods, they were categorized into the omnivorous dietary group (27). When examining the 24HDR dietary intake data, we found one deviation from the dietary group categorization, in which one participant categorized as lacto-ovo-vegetarian reported fish consumption once. This information was additionally cross-checked with their responses to the dietary screener assessing food group consumption in the past 6 months, and no inconsistency with the dietary practice was observed; thus, the participant was not re-categorized.

A total of 244 youths were recruited. Nine participants were excluded, whereof seven due to dietary practice ineligibility [six self-identified a flexitarian diet with limited intake of all animal-sourced foods (27) and one self-identified a pescatarian diet but contradicted this in the dietary screener reporting consuming meat], and two participants who only completed one out of four 24HDR’s.

### 2.5 Anthropometric measurements

At the research facility, weight was measured to the closest 0.1 kg while in light clothing and no shoes on using a Beurer 180BF digital scale (Beurer GmbH, Germany), and height was measured to the closest 1 mm by a wall-mounted stadiometer (Hyssna M, Sweden). Body mass index (BMI) was calculated, body weight (kg)/height (m^2^).

### 2.6 Assessment of dietary intake, 24-h dietary recalls

Dietary intake data was obtained through four non-consecutive web-based 24HDR’s (maximum of two were from a weekend). The first 24HDR was completed at the research facility as an interview following the Multiple Pass Method (28). Participants were asked to recall their complete dietary intake (including supplements) from the previous day. Probing questions were asked about time and place of food intake, and intake of commonly forgotten foods (e.g., beverages, condiments, sweets, snacks). Simultaneous to the interview, the interviewer entered the intake into a web-based dietary assessment program, Nutrition Data (Nutrition Data Sweden AB, Sweden). The program was connected to the Swedish Food Composition database (version 2023 06 13, Swedish National Food Agency) which consisted of 2,300 foods. Additionally, some items had been nutritionally calculated using brand product information. Following the visit, participants were asked to complete three self-administered 24HDR on different non-consecutive days in the web-based dietary assessment program. They received unannounced messages (text and email) asking them to complete a 24HDR. They received a maximum of three reminders per recall. Participants completed all their recalls within 7 weeks.

In the web-based program portion sizes could be reported in household measurements (teaspoon, tablespoon, deciliter), predefined quantities (e.g., slice, piece) or in weight (gram). Participants were provided a portion guide booklet developed by the Swedish National Food Agency [published 2010, Uppsala, Sweden (29)] and instructed to use it to estimate portions. The booklet contained 24 food photo series (e.g., cooked spaghetti, bolognese, rice, cereal etc.), each with 5–6 portion sizes per food. The original booklet did not contain photos for some commonly eaten snack foods. Therefore, prior to this study commencing, portion size photos were created, using a standardized method (30), for seven foods (nuts and dried fruit, candy, potato chips, popcorn, ice-cream, chopped tomatoes, and chopped carrots). The photos were added to the booklet, resulting in portion size photos for 31 different foods.

In the web-based program, participants were instructed to report separate food items (‘spaghetti’, ‘bolognese’) instead of composite meals (‘spaghetti with bolognese’). Additionally, recipes with specified ingredients and quantities could be entered into the program by the participants. If specific items could not be found in the program, participants were instructed to select nutritionally similar replacement foods (e.g., new plant-based soy meat analog could be replaced with a similar soy-based meat analog available in the program), or they could describe the item in an open notes section. All items specified in the open notes section were reviewed by the first author and subsequently entered into the recall. Either a nutritionally similar replacement food from the database was selected, or the nutritional information of the specific food item was obtained from the brand and thereafter entered into the program (e.g., organic plant-based dairy substitutes).

In this study, free sugars was defined according to the World Health Organizations definition (31), i.e., sugars from all foods which contain added sugars, as well as sugars which are naturally present in honey, syrups, fruit juice, and fruit juice concentrate. Content of sugars in foods was automatically calculated using the Swedish Food Composition database.

### 2.7 Consumption frequency of food groups

To assess the consumption frequency of food groups, in the web-based questionnaire participants were asked to fill in a dietary screener which assessed how often they had consumed 30 different food groups in the past 6 months. The 10 predefined frequencies ranged from ‘never/rarely’ to ‘6+ times per day’, see Supplementary Table 1. In this study, the original dietary screener, ‘MyFoodMonth 1.1’ (26), which was designed to capture intake frequencies for some key food groups consumed in Norway, and to reflect intakes of some nutrients (i.e., calcium, iodine), was translated from Norwegian to Swedish. Additionally, the questionnaire was revised to reflect food groups commonly consumed in Sweden and among individuals with plant-based diets. Thus, resulting in 30 food groups instead of 33 which the original dietary screener consists of.

## 3 Data processing and statistical analyses

### 3.1 Categorization of food groups

Food and drink items reported in the 24HDR were categorized into overall food categories based on nutritional similarity (‘fruits, berries, and vegetables’, ‘cereals and grains’) or function of the foods (‘plant-based meat analogs’, ‘sauces, dressings, and mayonnaise’). Subsequently, to distinguish between foods with differing nutritional characteristics within some of the overall food categories (e.g., ‘refined grain products’ and ‘whole grain products’) food groups were formed. Whole grain products were categorized as having >50% whole grains (dry weight) (32), which was determined by using the Swedish Food Composition database or brand product information. Description of foods included in each overall food category and food group is available as Supplementary Table 2.

For composite meals reported (e.g., ‘cheese hamburger’, ‘pizza’) more than 95% were disaggregated into separate foods (e.g., ‘burger bun’, ‘beef burger’, ‘tomatoes’) by the first author. This was done by using standardized ingredients and proportions available from the Swedish Food Composition database, whereof quantities were adapted to the portion reported. For brand specific composite meals reported (e.g., a frozen pasta meal), a standard and representative recipe was developed by using the product ingredient list and proportion of ingredients as to meet the nutritional information of the food. Some composite meals reported could not be disaggregated and were therefore grouped into the food category ‘composite foods’ (e.g., ‘spring rolls’, ‘dumplings’). This food category constituted <2% of the total mean EI per dietary group and was not included in the analyses of usual intakes of food groups.

### 3.2 Evaluation of misreporting of dietary intake

Misreporting of EI was evaluated for each participant using Goldberg cut-offs (33). Average reported EI (crude data) below, within, and above the cut-offs based on the participants physical activity level (PAL) was defined as under-, acceptable-, and over-reported, respectively. Basal metabolic rate was calculated for each participant using Henrys equations (34). Data is presented for descriptive purposes and in this study no participants were excluded based on misreporting.

#### 3.2.1 Assessment and categorization of physical activity level

The participants PAL (35) was assigned using questionnaire data which assessed the self-reported frequency of exercise per week in the last 6 months. Category of PAL was assigned as follows; PAL 1.4 = less than 1 times/week of moderate intensity; PAL 1.55 = 1–2 times/week of moderate intensity; PAL 1.7 = 3–4 times/week of moderate intensity; PAL 1.8 = 5–6 times/week of moderate intensity; PAL 2.0 = ≥6 times/week of moderate intensity and 1–2 times/week of vigorous intensity.

### 3.3 Estimation of usual dietary intakes

Usual individual dietary intakes were estimated for food groups, energy, macronutrients, whole grain, and salt using the Multiple Source Method (MSM) (36). The MSM is a two-part regression model which uses short-term dietary intake data, in the present study from the 24HDR’s, to account for within-and between person variation in intakes and as a result usual individual daily intake can be estimated. The statistical analyses were run using the web-based program (MSM analysis version 19Nov2009).^1^ First, the probability of consumption on a random day was estimated using a logistic regression, followed by estimation of the quantity consumed on a consumption day using a linear regression. Subsequently, the probability of consumption and quantity consumed on a consumption day are multiplied, resulting in an estimate of individual usual daily intake of the nutrient or food group. The statistical method has been described in detail elsewhere (37, 38). For the estimates of usual intake of food groups, consumption frequency, which was obtained from the dietary screener, was added as a covariate to the MSM-regression models. This improves the probability estimates. The consumption frequency from the dietary screener was converted to daily intakes. For example, if a participant reported consuming a food group once a week, the frequency was converted to 0.14 times/day, and if they reported consuming a food group 2–3 times a day the frequency was converted to 2.5 times/day etc. The dietary screener did not assess consumption frequency for all the specific food groups categorized in this study, for example vegetable and seed oil, refined grain products, and eggs. Therefore, consumption frequency was only added as a covariate in the MSM-models for 21 of the food groups.

Sensitivity analyses were run for food group intake, with adjustment for age and sex as covariates into the MSM-models. The covariates were selected based on theory. Furthermore, to account for differences in usual intakes of food groups relative to EI, the energy-density of food group intake was calculated for each individual, i.e., grams per megajoule (g/MJ) (39).

### 3.4 Adherence to the FBDG’s by NNR2023

To evaluate adherence to the FBDG’s the estimated usual intakes were compared to the quantitative FBDG’s by NNR2023 (7). To evaluate adherence to the FBDG of low-fat milk and dairy products, intakes of milk and yoghurt (≤1.5% fat) and low-fat dairy products (including cheese [≤17% fat] which had been converted to milk equivalents using a yield factor of 10) were included. Whole grain intake was automatically calculated using the Swedish Food Composition database or by brand product information.

### 3.5 Statistical analyses for comparison of dietary intakes between dietary groups

For categorical variables, differences between dietary groups were assessed using crosstabulation with Chi-Square or Fischer’s Exact test. Variables were assessed for normal distribution by visual inspection of histograms and Q-Q plots and the Shapiro Wilks test. For normally distributed continuous variables (age and BMI) the parametric One-Way ANOVA was used to test for differences between dietary groups. Dietary intake variables were mostly right skewed therefore the non-parametric Kruskal-Wallis One-Way ANOVA was used to test for differences between dietary groups. For all tests, the Bonferroni *post-hoc* test was applied to correct for multiple analysis, and statistical significance was accepted as two-sided adjusted *p*-value of <0.05. All dietary intake results are expressed as median value with 25th and 75th percentile, and number and percentage (%) for adherence to the FBDG’s. To allow for comparability with other studies, usual dietary intakes of food groups (g/d and g/MJ), energy, macronutrients, whole grain, and salt are presented with mean ± SD values in Supplementary material. Statistical analyses were performed using IBM SPSS version 29 (IBM Corp., Armonk, NY, United States).

## 4 Results

### 4.1 Participant characteristics

Participant characteristics are presented in Table 1. The total sample (*n* = 235) consisted of 60 vegans, 59 lacto-ovo-vegetarians, 55 pescatarians, and 61 omnivores. Of the total sample, 78% were female, mean age of 22 ± 2 years, and mean BMI of 23 ± 3 kg/m^2^, with no difference between dietary groups. Omnivores had the highest proportion (52%) with a PAL corresponding to a vigorous physical activity level (≥1.8) which differed from vegans (25%) and lacto-ovo-vegetarians (22%), *p* < 0.001. All four 24HDR’s were completed by 99% of the sample, and no difference was observed between dietary groups for percentage of recall days completed from a weekday or weekend (Table 1). Of the reported average EI in the 24HDR’s, 7% of the total sample’s average intakes were categorized as under-reported and none as over-reported, and it did not differ between dietary groups (Table 1).

**Table 1:** Participant characteristics of youth aged 16 to 24 years in Sweden adhering to vegan, lacto-ovo-vegetarian, pescatarian or omnivorous diets (*n* = 235).

	Total sample (*n* = 235)		Vegan (*n* = 60)		Lacto-ovo-vegetarian (*n* = 59)		Pescatarian (*n* = 55)		Omnivore (*n* = 61)		
Participant characteristics	Mean	SD	Mean	SD	Mean	SD	Mean	SD	Mean	SD	*p*-value
Age (years)^*^	22	2	22	2	22	2	21	2	21	2	0.05
BMI (kg/m^2^)^*^	23	3	22	3	22	2	22	3	23	3	0.07
	N	%	N	%	N	%	N	%	N	%	
**Sex**											0.11
Female^†^	183	78	45	75	49	83	47	86	42	69	
Male^†^	52	22	15	25	10	17	8	14	19	31	
**Use of tobacco products** ^‡^											
Snuff use^†^	66	28	14	23	19	32	17	31	16	26	0.68
Cigarette use^†,||^	40	17	11	19	12	20	9	17	8	13	0.74
**Physical activity level**											**0.01**
Low activity, PAL 1.4^†,¶^	14	6	8	13	4	7	1	2	1	2	
Moderate activity, PAL 1.55–1.7^†,¶^	144	61	37	62	42^a^	71	37	67	28^b^	46	
Vigorous activity, PAL 1.8–2.0^†,¶^	77	33	15^a^	25	13^a^	22	17	31	32^b^	52	
**Parental educational level**											
Mother, ≥3 years university education^†^	158	67	35	58	41	69	39	71	43	70	0.42
Father, ≥3 years university education^†^	120	51	29	48	29	49	34	62	28	46	0.33
**Misreporting of intake in the 24HDR’s** ^§^											0.39
Under reported intake^†^	17	7	2	3	5	8	4	7	6	10	
Acceptable reported intake^†^	218	93	58	97	54	91	51	93	55	90	
**Food intake day**											0.85
Weekday (Mon-Thur)^†^	621	66	163	68	156	66	145	67	157	64	
**24HDR, completed**											
Recall day 1^†^	235	100	60	100	59	100	55	100	61	100	n/a
Recall day 2^†^	235	100	60	100	59	100	55	100	61	100	n/a
Recall day 3^†,**^	234	99.6	60	100	59	100	54	98.2	61	100	0.23
Recall day 4^†,**^	232	98.7	59	98.3	58	98.3	54	98.2	61	100	0.71

SD, Standard deviation. BMI, Body mass index. PAL, Physical activity level. 24HDR, 24-h dietary recall. *Test for difference between groups using one-way ANOVA with Bonferroni *post-hoc* test. ^†^Test for difference was assessed by cross-tabulation using Pearson Chi-Square test or Fischer’s Exact Test, the percentage is shown within group. Unlike letters (^a,b^) in the same row indicate between which dietary groups there is a difference. ^‡^Snuff user and cigarette user were categorized as ‘never’ or ‘rarely/sometimes/daily’ according to their responses in the web-based questionnaire assessing snuff/cigarette use the past six months. ^||^Missing values for smoking (*n* = 4). ^¶^PAL1.4 = <1 times/week of moderate intensity; PAL 1.55 = 1–2 times/week of moderate intensity; PAL 1.7 = 3–4 times/week of moderate intensity; PAL 1.8 = 5–6 times/week of moderate intensity; PAL 2.0 = ≥6 times/week of moderate intensity and 1–2 times/week of vigorous intensity. ^§^Misreporting was evaluated based on average energy intake using Goldberg cut-offs (see methods for detailed description), and none had energy intakes categorized as over-reported. ^**^Values are given with one decimal place for meaningful values. Significance level was accepted as two-sided, adjusted, *p*-value of < 0.05 for all tests, and bolded *p*-values indicate significant difference between groups.

### 4.2 Food group intake among youth adhering to plant-based or omnivorous diets

Usual daily intakes of food groups (g/d and g/MJ) are presented as median value with 25th and 75th percentiles in Tables 2, 3.

**Table 2:** Absolute (g/d) usual daily intakes of food groups (median and 25th, 75th percentile) among youth aged 16 to 24 years in Sweden adhering to vegan, lacto-ovo-vegetarian, pescatarian or omnivorous diets (*n* = 235).

Food group	Total sample (*n* = 235)		Vegan (*n* = 60)		Lacto-ovo-vegetarian (*n* = 59)		Pescatarian (*n* = 55)		Omnivore (*n* = 61)		
Absolute usual daily intake, g/d	Median	25th, 75th	Median	25th, 75th	Median	25th, 75th	Median	25th, 75th	Median	25th, 75th	*p*-value^*^
Plant-based foods											
Vegetables, root vegetables, and mushrooms (all types)^†^	211	153, 259	218	164, 264	215	153, 268	184	144, 247	212	150, 256	0.47
Fruits and berries^‡^	102	55, 175	116	37, 200	109	61, 168	91	52, 158	98	55, 175	0.77
Fruit and vegetable juice	8	1, 46	8	2, 52	22	6, 62	7	1, 21	6	0, 36	0.05
Potatoes, plain	22	8, 47	23	13, 41	38^a^	10, 70	23^a^	13, 44	0^b^	0, 48	**<0.001**
Fried potatoes and potato dishes^§^	23	0, 40	9^a^	0, 34	0^a^	0, 35	28	0, 43	28^b^	18, 50	**<0.001**
Whole grain products^¶^	43	19, 74	41	24, 59	41	17, 67	50	18, 81	57	20, 99	0.13
Refined grain products	191	148, 253	221^a^	170, 280	200	135, 258	177^b^	148, 209	183	148, 242	**0.012**
Legumes	33	15, 59	83^a^	60, 120	36^b^	21, 53	32^b^	27, 38	10^c^	6, 14	**<0.001**
Nuts and seeds^||^	9	2, 20	13^a^	7, 24	10	3, 19	8^b^	1, 17	4^b^	1, 19	**0.002**
Vegetable and seed oils	7	4, 12	8^a^	5, 15	9	4, 12	8	4, 12	5^b^	3, 10	**0.016**
Plant-based meat analogs	52	19, 95	106^a^	82, 143	72^b^	44, 107	40^c^	23, 61	0^d^	0, 0	**<0.001**
Plant-based dairy substitutes	95	15, 171	184^a^	144, 279	116^b^	36, 171	33^c^	4, 106	20^c^	1, 82	**<0.001**
Animal-sourced foods											
Red meat and poultry (all types, including processed)	0	0, 79	0^a^	0, 0	0^a^	0, 0	0^a^	0, 0	141^b^	114, 175	**<0.001**
Fish, seafood, and fish products (all types)	0	0, 21	0^a^	0, 0	0^a^	0, 0	27^b^	6, 50	17^b^	5, 41	**<0.001**
Egg (all types)	0	0, 32	0^a^	0, 0	0^b^	0, 38	25^b^	0, 42	27^b^	0, 38	**<0.001**
Milk and dairy products^††^	144	0, 262	0^a^	0, 0	166^b^	91, 255	213^b^	142, 283	219^b^	135, 402	**<0.001**
Butter and margarine^§^	9	5, 13	8	3, 12	8	4, 14	10	6, 16	8	5, 12	0.20
Sweets and snack foods and beverages											
Candy and chocolate products^§^	14	6, 27	7^a^	2, 18	15^b^	8, 22	16^b^	9, 32	23^b^	6, 34	**<0.001**
Cakes, baked goods, sweet snack bars^§^	31	16, 46	40^a,c^	27, 48	23^b^	11, 35	35^c^	11, 49	27^b,c^	17, 41	**<0.001**
Ice-cream and cream-based puddings^§^	3	0, 22	0^a^	0, 16	8^b^	6, 23	3^a, b^	2, 20	0^a,c^	0, 23	**<0.001**
Salted snacks^§^	11	5, 19	12	7, 21	11	4, 20	10	0, 16	11	4, 23	0.19
Sugar sweetened beverages	51	9, 97	20	6, 120	22	9, 128	71	37, 97	46	0, 73	0.09
Alcoholic beverages	59	18, 150	46	17, 131	117^a^	0, 208	73	19, 129	33^b^	21, 118	**0.02**

g/d, gram per day. Usual intakes were calculated by the Multiple Source Method using repeated 24-h dietary recalls (between 2–4 days) and consumption frequency as a covariate (except for the food groups vegetable and seed oils, refined grains, and eggs since consumption frequency was not assessed). *For all variables, Kruskal-Wallis One-Way ANOVA was used test for differences between the dietary groups with Bonferroni *post-hoc* test to adjust for multiple comparisons. Bolded *p*-values indicate significant difference between the groups in the *post-hoc* test and unlike letters (^a,b,c,d^) in the same row indicate between which groups there is a difference. ^†^Includes processed vegetable products and excludes potatoes and legumes. ^‡^Includes fresh, frozen, canned, and dried fruit/berries, and excludes fruit juices (separate group) and fruit/berry jams, marmalades, and compotes. ^§^Includes vegan alternatives. ^¶^Whole grain products are defined as having > 50% (dry weight) whole grains (32). ^||^Includes salted nuts/seeds. ^††^Includes cheese and cheese products and excludes dairy ice-cream (ice-cream and cream-based pudding group) and butter (butter and margarine group). For details of foods included in each food group, refer to Supplementary Table 2. *p*-value of < 0.05 (two-sided) was accepted as statistically significant.

**Table 3:** Energy-density (g/MJ) of usual daily intakes of food groups (median and 25th, 75th percentile) among youth aged 16 to 24 years in Sweden adhering to vegan, lacto-ovo-vegetarian, pescatarian or omnivorous diets (*n* = 235).

Food group	Total sample (*n* = 235)		Vegan (*n* = 60)		Lacto-ovo-vegetarian (*n* = 59)		Pescatarian (*n* = 55)		Omnivore (*n* = 61)		
Energy-density of usual daily intakes, g/MJ	Median	25th, 75th	Median	25th, 75th	Median	25th, 75th	Median	25th, 75th	Median	25th, 75th	*p*-value^*^
Plant-based foods											
Vegetables, root vegetables, and mushrooms (all types)^†^	22	17, 28	23	19, 29	22	17, 29	22	17, 28	20	17, 28	0.28
Fruits and berries^‡^	11	6, 18	13	3, 22	12	6, 17	10	6, 17	10	6, 18	0.67
Fruit and vegetable juice	1	0, 5	1	0, 5	2^a^	1, 6	1	0, 2	1^b^	0, 3	**0.04**
Potatoes, plain	3	1, 5	3	1, 4	4^a^	1, 7	3^a^	2, 5	0^b^	0, 5	**<0.001**
Fried potatoes and potato dishes^§^	2	0, 5	1^a^	0, 4	0^a^	0, 4	3	0, 5	3^b^	2, 5	**<0.001**
Whole grain products^¶^	5	2, 8	5	3, 7	4	1, 8	6	2, 10	6	2, 9	0.23
Refined grain products	21	17, 26	24^a^	18, 29	21	17, 27	20^b^	16, 22	20^b^	14, 24	**0.004**
Legumes	4	1, 6	9^a^	6, 12	4^b^	3, 6	4^b^	3, 5	1^c^	1, 1	**<0.001**
Nuts and seeds^||^	1	0, 2	1^a^	1, 3	1	0, 2	1^b^	0, 2	0^b^	0, 2	**0.002**
Vegetable and seed oils	1	0, 1	1^a^	1, 2	1^a^	1, 1	1	0, 1	1^b^	0, 1	**0.001**
Plant-based meat analogs	6	2, 10	12^a^	9, 15	8^b^	5, 12	4^c^	3, 6	0^d^	0, 0	**<0.001**
Plant-based dairy substitutes	10	2, 19	20^a^	13, 34	12^b^	4, 21	4^c^	0, 12	2^c^	0, 8	**<0.001**
Animal-sourced foods											
Red meat and poultry (all types, including processed)	0	0, 8	0^a^	0, 0	0^a^	0, 0	0^a^	0, 0	16^b^	11, 18	**<0.001**
Fish, seafood, and fish products (all types)	0	0, 2	0^a^	0, 0	0^a^	0, 0	3^b^	1, 6	1^b^	0, 5	**<0.001**
Egg (all types)	0	0, 3	0^a^	0, 0	0^b^	0, 4	3^b^	0, 4	3^b^	0, 4	**<0.001**
Milk and dairy products^††^	15	0, 27	0^a^	0, 0	20^b^	11, 26	23^b^	15, 33	23^b^	14, 39	**<0.001**
Butter and margarine^§^	1	1, 1	1	0, 1	1	1, 1	1	1, 2	1	1, 1	0.06
Sweets and snack foods and beverages											
Candy and chocolate products^§^	2	1, 3	1^a^	0, 2	2^b^	1, 3	2^b^	1, 3	2^b^	1, 4	**<0.001**
Cakes, baked goods, sweet snack bars^§^	3	2, 5	4^a^	3, 6	3^b^	1, 4	4	2, 5	3^b^	2, 4	**<0.001**
Ice-cream and cream-based puddings^§^	0	0, 2	0^a^	0, 2	1^b^	1, 2	0^a,b^	0, 2	0^c^	0, 2	**<0.001**
Salted snacks^§^	1	1, 2	1	1, 2	1	0, 2	1	0, 2	1	0, 2	0.32
Sugar sweetened beverages	5	1, 11	2	1, 12	2	1, 13	8^a^	5, 11	5^b^	0, 7	**0.034**
Alcoholic beverages	6	2, 16	5	2, 13	12^a^	0, 23	9	2, 17	4^b^	2, 11	**0.009**

g/MJ, gram per megajoule. Usual daily intakes were calculated by the Multiple Source Method using repeated 24-h dietary recalls (between 2–4 days) and consumption frequency as a covariate (except for the food groups vegetable and seed oils, refined grains, and eggs since consumption frequency was not assessed). *For all variables, Kruskal-Wallis One-Way ANOVA was used to test for differences between dietary groups with Bonferroni *post-hoc* test to adjust for multiple comparisons. Bolded *p*-values indicate significant difference between the groups in the *post-hoc* test and unlike letters (^a,b,c^) in the same row indicate between which groups there is a difference. ^†^Includes processed vegetable products and excludes potatoes and legumes. ^‡^Includes fresh, frozen, canned, and dried fruit/berries, and excludes fruit juices (separate group) and fruit/berry jams, marmalades, and compotes. ^§^Includes vegan alternatives. ^¶^Whole grain products are defined as having > 50% (dry weight) whole grains (32). ^||^Includes salted nuts/seeds. ^††^Includes cheese and cheese products and excludes dairy ice-cream (ice-cream and cream-based pudding group) and butter (butter and margarine group). For details of foods included in each food group, refer to Supplementary Table 2. *p*-value of < 0.05 (two-sided) was accepted as statistically significant.

#### 4.2.1 Plant-based foods

For overall usual intakes of F&V (310 g/d, not including F&V juice), potatoes (50 g/d), and whole grain products (43 g/d), there were no differences between dietary groups. All plant-based dietary groups had higher usual intakes of legumes and plant-based meat analogs compared to omnivores, with vegans having the highest intakes. Furthermore, usual intakes of plant-based dairy substitutes were significantly higher among vegans (184 g/d) and lacto-ovo-vegetarians (116 g/d) compared to pescatarians and omnivores (33 and 20 g/d, *p* < 0.001). Moreover, vegans had higher usual intakes of refined grain products compared to pescatarians (221 vs. 177 g/d, *p* = 0.007), higher intakes of nuts and seeds compared to pescatarians and omnivores (13 vs. 8 and 4 g/d, *p* = 0.002), and higher intakes of vegetable oil compared to omnivores (8 vs. 5 g/d, *p* = 0.014). Lacto-ovo-vegetarians (38 g/d) and pescatarians (23 g/d) had higher usual intakes of potatoes (plain) compared to omnivores (0 g/d, *p* < 0.001), while omnivores (28 g/d) had higher intakes of ‘fried potatoes and potato dishes’ compared to vegans and lacto-ovo-vegetarians (9 and 0 g/d, *p* < 0.001; Table 2).

For intakes of plant-based foods based on energy-density (g/MJ), the findings remained mostly consistent with the absolute intakes (g/d). However, we found significant differences between dietary groups for intakes (g/MJ) of fruit juice (higher among lacto-ovo-vegetarians vs. omnivores), refined grain products (higher among vegans vs. both pescatarians and omnivores), and vegetable oil (higher among both vegans and lacto-ovo-vegetarians vs. omnivores; Table 3).

#### 4.2.2 Animal-sourced foods

No difference was found in usual intakes of dairy products and eggs, for either absolute intake (g/d) or intake based on energy-density (g/MJ), among consuming groups of dairy products and eggs (all dietary groups except vegans; Tables 2, 3). For fish and seafood, no difference was found in usual intakes (g/d and g/MJ) between pescatarians and omnivores. The omnivores had a usual intake of 141 g/d red meat and poultry (all types).

#### 4.2.3 Sweets and snack foods and beverages

For overall usual intakes of ‘sweets and snack foods’ (72 g/d) and sugar sweetened beverages (51 g/d) no differences were found between dietary groups (Table 2). However, differences were found in the usual intakes of specific food groups of ‘sweets and snack foods’. For ‘candy and chocolate products’ vegans had the lowest intakes compared to all other dietary groups (vegans 7 g/d, lacto-ovo-vegetarians 15 g/d, pescatarians 16 g/d, omnivores 23 g/d, *p* < 0.001). For ‘cakes, baked goods, and sweet snack bars’ vegans (40 g/d) had higher intakes compared to lacto-ovo-vegetarians (23 g/d) and omnivores (27 g/d; both, *p* < 0.001), while pescatarians (35 g/d) had higher intakes compared to lacto-ovo-vegetarians (*p* = 0.03). For ‘ice-cream and cream-based puddings’ lacto-ovo-vegetarians (8 g/d) had higher intakes compared to vegans and omnivores (both, 0 g/d), and pescatarians (3 g/d) had higher intakes compared to omnivores (*p* < 0.001). For alcoholic beverages, lacto-ovo-vegetarians had higher usual intakes compared to omnivores (117 vs. 33 g/d, *p* = 0.02).

For intakes based on energy-density (g/MJ), the findings remained mostly consistent with the absolute intakes (g/d) except for ‘cakes, baked goods, and sweet snack bars’, which no longer differed between pescatarians and lacto-ovo-vegetarians. However, for usual intakes of sugar sweetened beverages based on energy-density, pescatarians had higher intakes compared to omnivores (*p* = 0.022; Table 3).

#### 4.2.4 Sensitivity analyses for food group intake, adjustment for age and sex

In the sensitivity analyses performed in the MSM, adjusting food group intakes (g/d) for age and sex, the findings remained consistent with the unadjusted values, except for intakes of nuts and seeds, which no longer significantly differed between vegans and pescatarians (12 vs. 7 g/d, *p* = 0.05).

### 4.3 Energy and macronutrient intake among youth adhering to plant-based or omnivorous diets

Usual daily intakes of energy and macronutrients are presented as median value with 25th and 75th percentiles in Table 4. Omnivores had the highest usual EI (10 MJ/d) which differed from lacto-ovo-vegetarians and pescatarians (for both, 9 MJ/d, *p* = 0.016).

**Table 4:** Usual daily intake of energy, macronutrients, whole grain and salt (median and 25th, 75th percentile) among youth aged 16 to 24 year olds in Sweden adhering to vegan, lacto-ovo-vegetarian, pescatarian or omnivorous diets (*n* = 235).

		Total sample (*n* = 235)		Vegan (*n* = 60)		Lacto-ovo-vegetarian (*n* = 59)		Pescatarian (*n* = 55)		Omnivore (*n* = 61)		
Usual daily intake	Recommended daily intake^*^	Median	25th, 75th	Median	25th, 75th	Median	25th, 75th	Median	25th, 75th	Median	25th, 75th	*p*-value^†^
Energy, MJ/d	9.4 MJ (Female), 11.8 MJ (Male)^*^	9	8, 10	9	8, 10	9^a^	8, 11	9^a^	8, 10	10^b^	9, 11	**0.016**
Protein, g/d		74	61, 92	65^a^	52, 81	66^a^	58, 87	76^a^	63, 83	95^b^	75, 115	**<0.001**
Protein, g/kg^‡^	≥0.83 g/kg^§^	1.1	0.9, 1.4	1.1^a^	0.9, 1.3	1.1^a^	0.9, 1.3	1.1^a^	1.0, 1.4	1.4^b^	1.1, 1.7	**<0.001**
Protein, E%	10–20 E%	13	12, 16	12^a^	11, 13	12^a,b^	12, 14	14^b^	12, 15	16^c^	14, 17	**<0.001**
Carbohydrates, g/d		240	207, 276	263^a^	226, 299	232^b^	200, 265	228^b^	197, 247	243	213, 271	**<0.001**
Carbohydrates, E%	45–60 E%	46	42, 51	51^a^	47, 54	47^b^	43, 52	45^b,c^	41, 48	44^c^	40, 47	**<0.001**
Dietary fiber, g/d	≥25 g/d (Female), ≥35 g/d (Male)	28	23, 35	35^a^	30, 39	28^b^	24, 33	24^b^	21, 31	25^b^	19, 31	**<0.001**
Dietary fiber, g/MJ^‡^	≥3 g/MJ	3.1	2.6, 3.7	3.7^a^	3.3, 4.2	3.2^b^	2.7, 3.7	2.8^b^	2.2, 3.4	2.6^c^	2.1, 2.8	**<0.001**
Total sugars, g/d		79	66, 91	80	65, 89	77	69, 88	76	67, 88	83	65, 97	0.57
Total sugars, E%		14	12, 16	14	12, 17	14	12, 16	14	13, 16	14	11, 16	0.80
Free sugars, g/d^‖^		37	30, 45	36	28, 49	38	33, 43	38	30, 45	37	26, 48	0.84
Free sugars, E%^‖^	<10 E%	6	5, 8	6	5, 8	6	5, 8	6	5, 8	6	4, 8	0.35
Fat, total, g/d		92	78, 109	88^a^	75, 100	88^a^	71, 112	91	77, 108	102^b^	90, 113	**0.002**
Fat, total, E%	25–40 E%	38	35, 41	36^a^	32, 39	38	35, 42	40^b^	35, 42	39^b^	37, 42	**<0.001**
SFA, g/d		29	22, 37	21^a^	17, 24	27^b^	21, 37	32^b,c^	26, 38	36^c^	31, 42	**<0.001**
SFA, E%	<10 E%	12	9, 14	8^a^	7, 10	12^b^	9, 14	13^b,c^	11, 15	14^c^	12, 16	**<0.001**
MUFA, g/d		38	33, 45	38	31, 45	36^a^	30, 43	37	32, 44	42^b^	36, 46	**0.017**
MUFA, E%	10–20 E%	16	14, 17	15	14, 18	16	14, 17	16	14, 17	16	15, 17	0.76
PUFA, g/d		17	14, 22	23^a^	19, 26	16^b^	14, 21	16^b^	13, 19	15^b^	13, 17	**<0.001**
PUFA, E%	5–10 E%	7	6, 8	9^a^	8, 10	7^b^	6, 8	7^b^	6, 8	6^c^	5, 7	**<0.001**
Whole grain, g/d^¶^	≥90 g/d	44	26, 66	45	33, 64	43	24, 60	44	20, 67	48	27, 82	0.38
Salt, g/d^††^	<6 g/d	7	6, 9	8	6, 9	7	6, 8	7^a^	6, 8	8^b^	7, 9	**0.004**

MJ/d, Megajoule/day. SFA, Saturated fatty acids. MUFA, Monounsaturated fatty acids. PUFA, Polyunsaturated fatty acids. Usual daily intakes were calculated by the Multiple Source Method using repeated 24-h dietary recalls (between 2–4 days). ^*^Recommended dietary intake by NNR2023 for healthy 18–24 year olds, and recommended energy intake is based on a standard weight and physical activity level of 1.6. ^†^For all variables, Kruskal-Wallis One-Way ANOVA was used to test for differences between dietary groups with Bonferroni *post-hoc* test to adjust for multiple comparisons. Bolded *p*-values indicate significant difference between the groups in the *post-hoc* test, and unlike letters (^a,b, c^) in the same row indicate between which groups there is a difference. ^‡^Values are given with one decimal place for meaningful values. ^§^Recommended daily intake of protein in gram per kilogram body weight for adults ≥ 18 years, both sexes. ^‖^Free sugars was defined according to WHO’s definition (31), i.e., sugars from all foods which contain added sugars, as well as sugars which are naturally present in honey, syrups, fruit juice and fruit juice concentrate. ^¶^Whole grain content in foods was automatically calculated using the Swedish Food Composition database or by brand product information. ^††^Salt from foods only (including salt in the cooking method, e.g., ‘cooked pasta with salt’), and not including discretionary sources of salt reported in the 24HDR’s. *p*-value of < 0.05 (two-sided) was accepted as statistically significant.

For intakes of macronutrients based on percentage of energy (E%), omnivores had usual intakes of carbohydrates (including fiber) marginally below recommendations (44E%), and the intake was significantly lower compared to vegans (51E%) and lacto-ovo-vegetarians (47E%), *p* < 0.001. Further, all plant-based dietary groups had higher usual intakes of fiber (g/MJ) compared to omnivores, *p* < 0.001. However, both pescatarians (2.8 MJ/d) and omnivores (2.6 g/MJ) did not meet the recommendation for fiber according to energy density, ≥3 g/MJ. All dietary groups had usual intakes of free sugars (6E%) in line with the recommendation of <10E%. All dietary groups had usual intakes of protein and total fat within NNR2023 recommendations. Omnivores had higher usual intakes of protein (16E%) compared to all the plant-based dietary groups (vegans 12E%, lacto-ovo-vegetarians 12E%, pescatarians 14E%, *p* < 0.001). Vegans had the lowest usual intake of total fat (36E%), differing from both pescatarians and omnivores (40E% and 39E%, *p* < 0.001). All dietary groups except vegans (8E%) exceeded the recommendation of <10E% SFA (lacto-ovo-vegetarians 12E%, pescatarians 13E%, omnivores 14E%, *p* < 0.001). Moreover, all dietary groups had E% from monounsaturated and polyunsaturated fatty acids within recommendations, although all plant-based dietary groups had higher E% from polyunsaturated fatty acids compared to omnivores (*p* < 0.001). Omnivores had significantly higher usual intakes of salt (from foods only) compared to pescatarians (8 vs. 7 g/d, *p* = 0.004). Although, all dietary groups had usual intakes of salt that exceeded the recommendation of 6 g/d.

### 4.4 Adherence to the food-based dietary guidelines

The number and percentage of participants with usual intakes that meet the FBDG’s from the NNR2023 is presented in Table 5.

**Table 5:** Proportion of youth adhering to plant-based or omnivorous diets with usual intakes that meet the quantitative food-based dietary guidelines (FBDG) from the Nordic Nutrition Recommendations 2023 (NNR2023).

		Total sample (*n* = 235)		Vegan (*n* = 60)		Lacto-ovo-vegetarian (*n* = 59)		Pescatarian (*n* = 55)		Omnivore (*n* = 61)		
		% meeting recommendation^*^		% meeting recommendation		% meeting recommendation		% meeting recommendation		% meeting recommendation		
Food groups	FBDG by NNR2023	N	%	N	%	N	%	N	%	N	%	*p*-value^†^
Vegetables, fruits, and berries^*,‡^	500–800+ g/day	23	10	8	13	3	5	6	11	6	10	0.49
Whole grains^*,§^	≥90 g/day	24	10	1^a^	2	2^a^	3	7	13	14^b^	23	**<0.001**
Nuts^*,¶^	20–30 g/day	56	24	20	33	13	22	10	18	13	21	0.23
Vegetable oil^*^	≥25 g/day	10	4	5	8	2	3	2	4	1	2	0.20
Low-fat milk and dairy products^*,||^	350–500 g/day	26	11	0^a^	0	4^a, b^	7	8^b^	15	14^b^	23	**<0.001**
Fish (lean and oily fish)^*^	300–450 g/week	31	13	0^a^	0	0^a^	0	16^b^	29	15^b^	25	**<0.001**
Whereof oily fish^*,††^	≥200 g/week	20	9	0^a^	0	0^a^	0	15^b^	27	5^a^	8	**<0.001**
Red meat (including processed)^*^	<350 g/week	187	80	60^a^	100	59^a^	100	55^a^	100	13^b^	21	**<0.001**
Salt (foods only)^*^	<6 g/day	45	19	11	18	13	22	15	27	6	10	0.11

^*^ Adherence to the FBDG is based on individual usual intakes which were estimated by the Multiple Source Method using repeated 24-h dietary recalls (between 2–4 days) and consumption frequency (except for the food groups vegetable oil, low-fat milk and dairy, oily fish, and salt since consumption frequency was not assessed). Usual intakes were compared to the lower cut-off of the FBDG. ^†^For all variables, to test for differences between dietary groups crosstabulation with Fischer’s exact test was used. Bolded *p*-values indicate significant differences and unlike letters (a,b) in the same row indicate between which dietary groups there is a difference. ^‡^Not including potatoes, legumes, fruit/vegetable juices, or fruit jams/compotes/soups. ^§^Whole grain intake was calculated using the Swedish Food Composition database or by brand product information. ^¶^Includes salted nuts. ^||^Includes intakes of low-fat milk (≤1.5%), yoghurt (≤1.5%) and dairy products (incl. Low-fat cheese [≤17% fat], which was converted to milk equivalents using a yield factor of 10). ^††^At least 200 g of the recommended total fish intake should be from oily fish. *p*-value of < 0.05 (two-sided) was accepted as statistically significant.

Among the total sample, 10% met the FBDG of ≥500 g/d of F&V, 24% met the FBDG of ≥20 g/d nuts (including salted nuts), 4% met the FBDG of ≥25 g/d vegetable oil, and 19% met the FBDG of <6 g salt, with no significant difference between dietary groups. Vegans had the highest and lacto-ovo-vegetarians the lowest adherence to the FBDG for F&V (13% vs. 5%) and highest among vegans and lowest among omnivores for vegetable oil (8% vs. 2%), while vegans had the highest and pescatarians the lowest adherence to the FBDG for nuts (33% vs. 18%). For whole grains, 10% of the total sample met the FBDG of ≥90 g/d, with significantly higher adherence among omnivores (23%) compared to vegans and lacto-ovo-vegetarians (2 and 3%, *p* < 0.001).

Of the consuming dietary groups (excluding vegans), no difference was found for adherence to the FBDG of ≥350 g/d of low-fat milk and dairy products. Less than a third of pescatarians (29%) and omnivores (25%) had usual intakes which met the FBDG of 300–450 g/week of fish (total), with no difference between the two dietary groups. However, pescatarians had a higher adherence to the FBDG of ≥200 g/week of oily fish compared to omnivores (27% vs. 8%, *p* < 0.001). For red meat (including processed), 21% of the omnivores had intakes that met the FBDG of <350 g/week.

## 5 Discussion

### 5.1 Key findings of dietary intake among youth adhering to plant-based or omnivorous diets

In this study of 16 to 24 year olds in Sweden, youth adhering to plant-based diets had higher usual intakes of legumes and plant-based meat analogs compared to omnivores, with highest intakes among vegans. Additionally, vegans and lacto-ovo-vegetarians consumed more plant-based dairy substitutes compared to pescatarians and omnivores. Furthermore, differences were found for usual intakes (g/d and g/MJ) of refined grain products, nuts and seeds, and vegetable oil (highest among vegans), plain potatoes (highest among lacto-ovo-vegetarians), fried potatoes and potato dishes (highest among omnivores), and for food groups within the category of ‘sweets and snack foods’. Intakes of fruits and berries, vegetables, whole grain products, and overall intakes of ‘sweets and snack foods’ did not differ between the dietary groups. These findings remained mostly consistent when adjusted for sex and age. Most of the NNR2023 macronutrient recommendations were met across dietary groups, except for carbohydrates (below for omnivores), fiber (below for omnivores and pescatarians), and SFA (exceeded for lacto-ovo-vegetarians, pescatarians, and omnivores). We found no difference between the dietary groups for adherence to the FBDG’s for F&V, nuts, vegetable oil, salt, low-fat dairy (excluding vegans), and total fish (excluding vegans and lacto-ovo-vegetarians). Although, omnivores had a significantly higher adherence to the FBDG for whole grains compared to vegans and lacto-ovo-vegetarians, and pescatarians had a higher adherence to the FBDG for oily fish compared to omnivores. Nevertheless, the vast majority of the participants did not meet the NNR2023 FBDG’s, regardless of dietary practice.

### 5.2 Intakes of plant-based food groups

To meet energy and nutritional requirements when adhering to a plant-based diet, animal-sourced foods need to be replaced with other food groups. In this study, all plant-based dietary groups consumed more legumes and plant-based meat analogs compared to omnivores. Moreover, vegans consumed more refined grain products, nuts/seeds, vegetable oil, and plant-based dairy substitutes compared to omnivores and/or pescatarians, indicating that these food groups replaced animal-sourced foods. Our findings are somewhat similar with previous studies on children/adolescents in Germany (14) and youth in Norway (40) which also observed higher average intakes (g/d or g/MJ) of nuts and seeds, legumes, and plant-based meat and dairy alternatives among vegans compared to omnivores. A previous study of youth in Sweden (from the late 1990’s) also found that vegans consumed more nuts and seeds and legumes compared to omnivores of similar age (19, 41). However, we found no difference between the dietary groups for usual intakes of F&V which stands in contrast to the aforementioned studies on children/adolescents and youth adhering to plant-based or omnivorous diets, which observed higher average intakes (g/d or g/MJ) of fruits and berries and/or vegetables among vegans compared to omnivores (14, 19, 40, 41).

### 5.3 Replacement of animal-sourced foods among youth adhering to plant-based diets

#### 5.3.1 Plant-based meat analogs and legumes

The NNR2023 state that legumes should constitute a significant part of the diet since they are a source of protein, complex carbohydrates, dietary fiber, folate, zinc, and iron, as well as being low in SFA and have a low environmental impact (7). There is no dietary recommendation for intakes of plant-based meat analogs from the NNR2023.

In our study vegans had usual intakes (g/d and g/MJ, median value) of plant-based meat analogs (106 g/d, 12 g/MJ) somewhat lower than the omnivores intakes of red meat and poultry (141 g/d, 16 g/MJ). However, vegans consumed eight times more legumes compared to omnivores (83 g/d vs. 10 g/d). Furthermore, lacto-ovo-vegetarians and pescatarians consumed four-to-seven times the intake of plant-based meat analogs and three-fold the intake of legumes compared to omnivores. Thus, our findings suggest that the youth in this study who adhered to plant-based diets replaced animal-sourced foods (i.e., red meat and poultry) with plant-based meat analogs, and to a lesser extent with legumes. However, the youth adhering to plant-based diets in our study had much lower intakes of legumes and vegetables compared to what was observed among vegans in the late 1990’s who had average intakes of 255–352 g/d of legumes and 292–320 g/d of vegetables (median values) (42). In the late 1990’s plant-based meat analogs were less common. The growing variety and availability of plant-based meat analogs in recent years (23), may lead to current youth favoring these products as a replacement for animal-sourced foods over legumes, whole grain products, and vegetables. Nevertheless, in our study, the higher intake of legumes and plant-based meat analogs, and the absence of red or processed meat among youth eating plant-based diets demonstrated better alignment with the food and macronutrient recommendations compared to omnivores of similar age.

Replacing red meat with plant-based meat analogs can lower the environmental impact from dietary intake (3). Furthermore, if fortified they can provide equal or greater quantities of micronutrients, and simultaneously provide a more favorable nutritional content of SFA and dietary fiber compared to red and processed meat (3). However, not all plant-based meat analogs are fortified, and protein content of these products varies, and additionally there are concerns over their high sodium content and limited bioavailability of iron and zinc (20, 21). Furthermore, while legumes are nutrient dense, they lack some essential micronutrients found predominantly in red meat, poultry, and fish, including vitamin B_12_, vitamin D, iodine, and omega-3 fatty acids (43–45). Thus, youth eating plant-based diets, particularly vegan and lacto-ovo-vegetarian diets, need to plan their dietary intakes to ensure that they meet their requirements of micronutrients by other food sources or by use of appropriate supplementation.

#### 5.3.2 Dairy products and plant-based dairy alternatives

To ensure sufficient intake of calcium, iodine, and vitamin B_12_, NNR2023 recommend that fortified plant-based dairy products replace milk and dairy if intakes are less than 350 g/d (7). In this study, lacto-ovo-vegetarians (166 g/d, 20 g/MJ) and pescatarians (213 g/d, 23 g/MJ) had similar total dairy intakes to omnivores (219 g/d, 23 g/MJ), and few consumed low-fat dairy products which is recommended by NNR2023. Vegans reported intakes of plant-based dairy products (184 g/d, 20 g/MJ) somewhat similar to dairy intakes among omnivores, indicating that dairy is mostly replaced with plant-based alternatives. Also, in this study lacto-ovo-vegetarians appear to partially replace dairy products with plant-based dairy alternatives (116 g/d, 12 g/MJ).

Plant-based dairy substitutes have both nutritional benefits and shortcomings. They contain lower SFA (except for coconut-based products) and more fiber compared to milk and dairy products, although the protein and sugar content varies by product (22). A recent nutritional composition study of plant-based substitutes on the Swedish market showed that fortified plant-based substitutes of milk and yoghurt can provide similar micronutrient content as fortified dairy products (22). In Sweden, most plant-based milks are fortified with vitamin D, vitamin B_12_, calcium, and vitamin B_2_, and few are fortified with iodine (22). However, plant-based dairy alternatives for yoghurt, cheese, and cream are not as commonly fortified compared to milk alternatives. Furthermore, there are differences in fortification policies across Nordic countries which impact the nutritional equivalence of plant-based substitutes to dairy. The variation in fortification between products may negatively influence micronutrient intake if the plant-based diet is not well-planned and if dairy is replaced with unfortified plant-based dairy alternatives.

### 5.4 Intakes of sweets and snack foods

The NNR2023 recommend limited intakes of sweets and sugar sweetened foods/beverages as they contribute primarily to sugar, added fat, and energy, while providing minimal nutritional value (7). Dietary intakes of discretionary foods in the form of sweets and snack foods are relatively high among adults and young populations in Scandinavian countries (9, 46, 47), which was also observed in our study. Furthermore, we observed no difference between the dietary groups for overall usual intakes of ‘sweets and snack foods’ or absolute intakes (g/d) of sugar sweetened beverages. Although the types of sweets and snack foods consumed differed between the dietary groups. These results align with a similar study of youth in Norway who ate plant-based or omnivorous diets (*n* = 165, 16–24 years) (40). Our findings indicate that while youth adhering to plant-based diets increase their intakes of some plant-based foods compared to omnivores of similar age, their overall intakes of sweets and snack foods do not decrease. If sweets and snack foods displace healthy plant-based foods, it may present a challenge in meeting micronutrient recommendations from foods without exceeding energy requirements.

### 5.5 Intakes of energy, macronutrients and salt

We found that omnivores had higher usual EI (median value) compared to pescatarians and lacto-ovo-vegetarians. However, the omnivorous group had a higher percentage of males (though not significantly different), and a higher proportion reported vigorous physical activity, both of which are associated with greater energy needs.

Our results demonstrate that the usual intakes of protein (E%) and SFA (E%) decreased while the intakes of carbohydrates (E%) and fiber (g/MJ) increased between the dietary groups in parallel with the reduction of animal-sourced food groups included in the diet, and vegans were the only dietary group to have intakes of SFA within recommendations. These findings are in agreement with the overall findings of average mean intakes from previous cross-sectional studies of healthy children/adolescents (2–18 years, 30 studies) (16), Swedish youth (19) and adults (≥18 years, 141 studies) (18) adhering to vegan, lacto-ovo-vegetarian or omnivorous diets. Also, mostly in line with a similar study of youth in Norway eating plant-based (vegan, lacto-ovo-vegetarian, pescatarian, flexitarian) or omnivorous diets (40).

In our study, vegans had macronutrient intakes most aligned with the dietary recommendations. The omnivores had usual intakes of macronutrients least aligned with the dietary recommendations (i.e., SFA, carbohydrates, and fiber), which is partially explained by their lower intakes of plant-based foods (e.g., legumes) and higher intakes of animal-sources of protein (red meat and processed meat and high fat dairy products). All dietary groups had usual intakes (E%) of free sugars in line with the dietary recommendations (<10E%), which stands in contrast to the dietary intakes found among Swedish youth from the national food consumption survey (9.8-11E%) (48). A potential explanation for the lower intake of free sugars we found among the youth in the present study, is that their reported intakes of sugar sweetened beverages, a leading contributor to sugar intake, was half the amount compared to the reported intakes by Swedish youth in general (51 vs. 100 g/d, median value) (9, 48). Furthermore, intakes of free sugars may be slightly underestimated due to some foods in the food composition database being nutritionally calculated by brand product information, therefore the content of free sugars was not available. All dietary groups in this study exceeded the recommendation for salt, whereof only 19% of the youth had intakes of salt below 6 g/d. Intakes of salt are solely from foods and their preparation (e.g., ‘pasta cooked with salt’), thus usual intakes are likely underestimated as discretionary salt was not included. Further research is needed to explore the main dietary sources of energy, salt, macro- and micronutrients in the youths’ intakes, and identify strategies for promoting healthier dietary habits among youth.

### 5.6 Adherence to the FBDG’s for F&V and whole grains

A low adherence to the FBDG’s, particularly for F&V, whole grains, and nuts has been observed among both adolescents and adults in many European countries, including Sweden (9, 47, 49, 50). In this study, one in 10 had usual daily intakes of ≥500 g F&V and ≥90 g whole grains which is comparable to findings from the Swedish national food consumption surveys, where 10% of youth (17–18 years) and 17% of adults had daily intakes of F&V that met the FBDG, and ≤8% of youth and 12% of adults met the dietary recommendations for whole grains ((9, 25, 49). Vegans in this study had the highest proportion with usual intakes meeting the FBDG for F&V and nuts and seeds, as well as vegetable oil, indicating somewhat healthier food habits on individual level. Although, a previous study of young Swedish vegans (*n* = 30) from the late 1990’s found that 70% (21 out of 30) of the vegans met the recommended daily intake of 500 g F&V (excluding potatoes) compared to 3% of omnivores (1 out of 30) of similar age (41). Thus, our findings indicate that current youth eating plant-based consume lower intakes of F&V compared to vegans in the late 1990’s, likely as a result of the increased availability of different types of pre-made plant-based foods. Furthermore, in this study omnivores had a significantly higher adherence to the recommended intakes of whole grains compared to vegans and lacto-ovo-vegetarians, demonstrating that youth eating plant-based diets in this study consumed refined grain products more often than whole grain products. Whole grain products are more nutrient dense than refined grain products, and they provide some micronutrients which may be consumed in lower quantities when animal-sourced foods are excluded from the diet (including iron, zinc, selenium, and riboflavin (16, 17, 51)). Thus, replacing refined grain products with whole grain alternatives would support youth, particularly those eating plant-based, in meeting their requirements of micronutrients.

It might be expected that youth who adhere to plant-based diets increase their intakes of F&V as to meet nutritional requirements. However, factors including convenience, availability, price, and taste and sensory aspects are likely more influential in shaping youths’ food choices and consumption of F&V as well whole grain products (52–54). Further, whether they possess food-related competencies, including skills, knowledge and behaviors which can facilitate them in making food choices aligned with the dietary guidelines, i.e., food literacy competencies (55, 56). A recent study in Norway observed a positive association between youth’s food literacy (general nutrition knowledge and food skills) and diet quality, although the youth had food literacy levels categorized as moderate (57). As long-term low intakes of F&V and whole grains are associated with an increased risk for non-communicable diseases (cardiovascular disease, all-cause mortality, type-2 diabetes (58–60)), youth should be supported in increasing their intakes of these food groups and developing food literacy competencies necessary to consume healthy diets.

### 5.7 Strengths and limitations

To the best of our knowledge, this is the first study since the late 1990’s to assess the dietary intakes of youth in Sweden eating plant-based diets compared to omnivorous diets. The strength of this study is the comparable number of participants with similar characteristics within each dietary group. Furthermore, there was a high compliance to the 24HDR dietary assessments and less than 10% of the average reported intakes in the 24HDR’s were categorized as under-reported. Short-term dietary intake data may fail to capture foods consumed episodically and does not account for within-and between person variation. Thus, to account for this, usual daily intakes were estimated using the MSM which adjusts for variability in dietary intakes. Additionally, for food group intake, the short-term dietary intake data from the 24HDR’s was used in combination with consumption frequency in the MSM, which improves the estimates of usual daily intakes, particularly for food groups consumed episodically (37, 38). While the small sample size may affect the reliability of usual intake estimates by the MSM, the results were largely consistent with those based on average intakes (data not shown), except for some food groups less commonly reported in the 24HDR’s (e.g., ‘ice-cream and cream based puddings’ and ‘fried potatoes and potato dishes’). This suggests that the potential limitation of the sample size did not impact our overall findings of dietary intakes, but the MSM improved intake estimates for episodically consumed foods. Nevertheless, this study has limitations to be considered. First is the cross-sectional design and the use of convenience sampling, which resulted in a study sample with mostly female participants which impacts the generalizability of our results. Although, females are over-represented among plant-based consumers in previous literature (14, 57, 61), thus the sample recruited may be representative of youth populations consuming plant-based diets. Furthermore, although we performed sensitivity analyses for intakes of food groups (g/d), adjusting for sex and age—which supported our overall findings, due to the similarity in participant characteristics, potential differences related to sex and age may not have been detectable. Lastly, given the study topic and the recruitment methods employed (convenience and snowball sampling), the potential that the youth who were recruited in this study were more “health conscious” than youth in general needs to be acknowledged.

## 6 Conclusion

In conclusion, youth adhering to vegan, lacto-ovo-vegetarian, and pescatarian diets consume higher intakes of legumes and plant-based meat analogs compared to omnivores, suggesting that these food groups replace meat in the diet. Additionally, the highest intakes of several plant-based foods (legumes, nuts and seeds, refined grain products, plant-based meat and dairy alternatives) was observed among vegans. However, very few of the youth in this study had usual intakes that meet the recommended dietary intakes of F&V, nuts, vegetable oil, and whole grains, regardless of eating a plant-based or omnivorous diet. Although intake of energy and macronutrients are mostly in line with recommendations, youth face a challenge to reduce intakes of discretionary foods and consume enough fruits, berries, vegetables, nuts, and whole grain products. Thus, youth need support to better align with food recommendations if their potential for long-term individual health as well as planetary health is to be secured.

## Data Availability

The datasets presented in this article are not readily available because of ongoing analysis. However, data can be made available on reasonable request. Requests to access the datasets should be directed to isabelle.mulkerrins@gu.se.

## Footnotes

^1^ https://msm.dife.de/tps/en
