# Supplementary material for: Dietary intake among youth adhering to vegan, lacto-ovo-vegetarian, pescatarian or omnivorous diets in Sweden
Source: Front Nutr. 2025 Mar 10;12:1528252. doi: 10.3389/fnut.2025.1528252 (PMC11930832; doi:10.3389/fnut.2025.1528252)
Supplement: Supplementary file 1 [file Data_Sheet_1.pdf]

Supplementary material

Supplementary Table 1. Dietary screener used for assessment of consumption frequency of 30 food groups among youth adhering to vegan, lacto-ovo-vegetarian, pescatarian or omnivorous diets (n=235).

Supplementary Table 2. Description of food and drink items included in the food group intakes among youth aged 16 to 24 years in Sweden adhering to vegan, lacto-ovo-vegetarian, pescatarian or omnivorous diets (n=235).

Supplementary Table 3. Absolute (g/d) usual daily intake of food groups (mean±SD) among youth aged 16 to 24 years in Sweden adhering to vegan, lacto-ovo-vegetarian, pescatarian or omnivorous diets (n=235).

Supplementary Table 4. Energy-density (g/MJ) of usual daily intakes of food groups (mean±SD) among youth aged 16 to 24 years in Sweden adhering to vegan, lacto-ovo-vegetarian, pescatarian or omnivorous diets (n=235).

Supplementary Table 5. Usual daily intakes of energy, macronutrients, whole grain and salt (mean±SD) among youth aged 16 to 24 years in Sweden adhering to vegan, lacto-ovo-vegetarian, pescatarian or omnivorous diets (n=235).

Supplementary Table 1. Dietary screener used for assessment of consumption frequency of 30 food groups among youth adhering to vegan, lacto-ovo-vegetarian, pescatarian, or omnivorous diets (n=235).

**Question. How often have you eaten the following foods? Think back and answer how it has been the past six months.**
- Sweetened breakfast cereals e.g., cheerios, Special K, granola<br>- Unsweetened breakfast cereals e.g., muesli, oats, Weetabix, “rågfras [Swedish]”, “havreringar [Swedish]”<br>- Wholegrain bread and/or crisp bread<br>- Wholegrain pasta, rice, grains, e.g. whole grain bulgur, whole grain couscous<br>- White pasta, rice, grains<br>- Dairy products (e.g., yoghurt, milk, cheese, crème fraiche, cooking cream), all types<br>- Plant-based dairy alternatives (e.g., oat milk, soya yoghurt, oat crème fraiche), all types<br>- Butter, margarine, liquid cooking fats (including vegan alternatives)<br>- Vegetables (including salad, cabbage, root vegetables, mushrooms), all types. Not including potatoes and sweet potatoes<br>- Beans, lentils, chickpeas, green peas (including hummus, falafel, etc.), all types<br>- Tofu and/or tempeh<br>- Fruit and/or berries, all types (fresh, frozen)<br>- Potatoes or sweet potatoes (example cooked, mashed). Not including fried potatoes<br>- Potatoes fries or other fried potatoes<br>- Fish and seafood, all types<br>- Fishfingers, fish burgers, “fiskbullar [Swedish]”, or other types of premade fish-based products<br>- Chicken, turkey, duck<br>- Red meat (e.g., lamb, mince, steak, wild meat)<br>- Processed meat (e.g., hamburger, sausages, meatballs), all types<br>- Plant-based meat substitutes (e.g., burger, sausage, mince), all types. Not including tofu or tempeh<br>- Pizza, premade pies, frozen premade meals, premade sandwiches, including vegan alternatives, all types<br>- Unsalted nuts and seeds, all types<br>- Salted snacks (e.g., popcorn, chips, salted nuts), including vegan alternatives<br>- Candy and chocolate, including vegan alternatives<br>- Baked goods, cakes, cookies and biscuits, waffles etc., including vegan alternatives<br>- Ice-cream, including vegan alternatives<br>- Fruit juice, all types<br>- Sugar-sweetened soda, juice, energy drink or sweetened coffee drinks (e.g., Coca-Cola, PowerKing etc.)<br>- Water<br><br>**Response alternatives**<br>- Never/rarely<br>- 1 time per month<br>- 2-3 times per month<br>- 1 time per week<br>- 2-4 times per week<br>- 5-6 times per week<br>- 1 time per day<br>- 2-3 times per day<br>- 4-5 times per day<br>- 6+ times per day

**Question. How often do you drink alcoholic beverages (all types)? Think back and answer how it has been the past six months.**
**Response alternatives**<br>- Never<br>- One times a month (or less often)<br>- 1 time per week<br>- 2-3 times per week<br>- 4 times or more per week<br>- Daily

Dietary screener is a revised version of the original "MyFoodMonth 1.1". Revisions include translating from Norwegian to Swedish and condensing from 33 food groups to 30 food groups, reflecting food groups commonly consumed in Sweden and among individuals eating plant-based diets.

Supplementary Table 2. Description of food and drink items included in the food group intakes among youth aged 16 to 24 years in Sweden adhering to vegan, lacto-ovo-vegetarian, pescatarian or omnivorous diets (n=235).

**Overall food category**	**Food group**	**Description of food and drink items included**
Vegetables, fruit, and berries	Vegetables, root vegetables, and mushrooms (all types)	All types of raw, frozen, cooked, steamed vegetables and root vegetables, also including mushrooms and sprouts. Including vegetable products e.g., sundried tomatoes, roasted onion, kimchi, pickled onions, baba ghanoush, coleslaw, beetroot salad, pizza salad, vegetable-based burgers, crushed tomatoes, tomato paste, algae (seaweed products). **Excludes** green peas (in the legumes and pulses food group) and potatoes (separate category).
	Fruits and berries (all types)	All types of fruit and berries, including fresh, frozen, canned, and dried fruit or berries. **Excludes** fruit/berry jams, marmalades, and compotes (separate category) as well as fruit juices (separate category).
Fruit and vegetable juice	Fruit and vegetable juice	Fruit and vegetables juices e.g., apple juice, orange juice, pineapple juice, beetroot juice, carrot juice, lime juice and lemon juice. **Excludes** concentrated fruit juices and cordials with added sugar (in the sugar sweetened beverages category) and concentrated fruit juices and cordials with sweeteners (in the sugar free beverages category).
Potatoes	Potatoes, plain	Potatoes and sweet potatoes, boiled or baked with no added fat
	Fried potatoes and potato dishes[*]	All types of potatoes and sweet potatoes that have been fried or prepared in some manner, e.g., fries, potato patties, hashbrowns as well as potato dishes e.g., potato gratin, potato salads, mashed potatoes, cheese fries. *Includes vegan alternatives.*
Cereals and grains	Whole grain products[†]	Whole grain products of bread, crisp bread, pasta, noodles, rice, couscous, breakfast cereals, granola and oats. Also includes pseudocereals[‡] such as quinoa, amaranth, buckwheat.
	Refined grain products	Cereals and grains which were not whole grain (e.g., having ≤50% of dry weight from whole grains), including bread, bread products (e.g., pizza dough, croutons), pasta, noodles, rice, couscous, bulgur, breakfast cereals, rice cakes, crackers etc. **Excludes** sweet pastries, sweet baked goods and cakes (in the food group, *cakes, baked goods, sweet snack bars).*
Legumes	Legumes	Beans, lentils, and peas, including legume-based flour, e.g., chickpea flour, also includes the composite food “lentil soup”. Includes different types of legume-based products e.g., bean burgers, falafel, hummus.
Nuts and seeds	Nuts and seeds	All types of nuts and seeds, including salted versions as well as nut butter and seed butters (e.g., tahini), almond flour, coconut flour, hempseeds, as well as coconut shreds and coconut flakes.
Plant-based meat analogs	Plant-based meat analogs	All types of plant-based meat analogs (soy based, pea based, wheat-based [seitan], mycoprotein), tofu, tempeh, textured vegetable protein, and sandwich toppings e.g., vegetarian/vegan ham slices, vegan paté. Also includes the plant-based meat analog dish “soya bolognese”.
Plant-based dairy substitutes	Plant-based dairy substitutes	All types of plant-based dairy alternatives for milks, yoghurt, cream (including whipping cream, cooking cream as well as coconut cream), sour cream, crème fraiche. Includes products based on different main ingredient (e.g., soya, oat, coconut, rice, nuts). Also includes different types of plant-based cheeses.
Red meat and poultry (all types including processed) [¶]	Poultry and poultry products[¶]	All types of poultry and processed poultry-based products, e.g., chicken, turkey, duck, and products based on these, e.g., sausage, burgers, chicken wings, chicken nuggets, minced chicken, and sandwich toppings e.g., chicken slices, turkey slices etc.
	Red meat and red meat products[¶]	All types of red meat and processed red meat products, e.g., minced meat, steak, lamb, meatballs, sausages, bacon, hamburgers, ham slices, salami (sandwich toppings), pate, blood pudding, pulled pork, kebab. Also includes the meat-based dish “bolognese”.
Fish, seafood and fish products	Fish and seafood	All types of fish and shellfish including processed fish products, e.g., salmon, tuna, cod, herring, mackerel, sardines, prawns.
	Fish-based products	Fish fingers, fish burgers, caviar, seafood-based sauces (e.g., shrimp salad).
Egg (all types)	Egg (all types)	All types of egg and egg-based dishes, e.g. scrambled eggs.
Dairy products[§]	Milk and dairy products[§]	All types of dairy products of milk, yoghurt, sour milk, cheese, cooking and whipped cream, sour cream, crème fraiche, quark. Includes flavoured alternatives, sugar-sweetened alternatives, and products with different fat contents. Includes all types of cheese including cheese products such as melted cheese dip, cottage cheese, cream cheese, and mozzarella sticks. Also includes dairy based yoghurts and drinks with added protein (but excludes concentrated milk protein powders “whey protein powder”, “casein protein powder” which are categorized in the protein supplements and bars category).
Sweets and snack foods	Candy and chocolate products[*]	All types of candy (incl. caramel and chocolate sauces and meringues) and chocolate (incl. chocolate spreads) products. *Includes vegan alternatives.*

	Cakes, baked goods, sweet snack bars*	All types of cakes (e.g., chocolate cake, carrot cake etc), cookies/sweet crackers (e.g., chocolate covered wafers), sweet snack bars (flapjacks, chocolate covered müsli bars), pastries (e.g., cinnamon buns, saffron buns, croissants), pancakes and waffles. *Includes vegan alternatives.*
	Ice-cream and cream-based puddings*	All types of ice-cream and sorbets, milkshakes, and sweetened cream-based puddings/desserts (e.g., sweet rice pudding). *Includes vegan alternatives.*
	Salted snacks*	All types of crisps including lentil crisps, popcorn, salted pretzel sticks, roasted/salted corn snacks (excludes salted nuts). *Includes vegan alternatives*.
Added fats	Vegetable and seed oils	All types of vegetable and seed oils, including coconut oil.
	Butter and margarine*	All types of butter and margarine as well as liquid-based margarines. *Includes vegan alternatives*.
Sugar-sweetened beverages	Sugar-sweetened beverages	All types of sugar-sweetened beverages incl. soda, caffeinated drinks (energy drink, iced tea) and concentrated juices or cordials.
Alcoholic beverages	Alcoholic beverages	All types of alcoholic beverages including low alcoholic equivalents.
**Food categories not included in analysis of usual intakes**		
Fruit/berry jams, compotes and soups	Fruit/berry jams, compotes and soups	All types of sugar-sweetened and low-sugar versions of jams, marmalades, apple sauce as well as sweet fruit/berry soups (e.g., "nyponsoppa", "blåbärssoppa" [Swedish], consisting of <10% berries/fruit).
Sauces, dressings and mayonnaise	Sauces, dressings and mayonnaise*	All types of sauces, dressings, and mayonnaise, including pesto, ketchup, sweet chili sauce, mango chutney, curry sauce, chili sauce, mayonnaise and mayonnaise-based sauces and dressings (e.g., aioli, bearnaise, hollandaise sauce, sriracha mayonnaise). *Includes vegan alternatives.*
Protein supplements and bars	Protein supplements and bars*	All types of protein powders as well as protein bars. Also includes essential amino acids and branched chain amino acids, as well as meal replacement powders or shakes. *Includes vegan alternatives.*
Unsweetened coffee and tea	Unsweetened coffee and tea	All types of unsweetened coffee and tea, e.g., brewed coffee, espresso, instant coffee, black tea, red tea, green tea, herbal tea.
Sugar free beverages	Sugar free beverages	All types of sugar free juice and cordials, soda and energy drinks, e.g., fun light, Zeroh!, cola zero, Nocco, Celsius, sugar free PowerKing.
Discretionary added sugar	Discretionary added sugar	All types of discretionary sources of added sugar, e.g., table sugar, brown sugar, syrup, honey, date syrup, agave syrup, maple syrup, dextrose, coconut sugar. *Not including sugar in foods/dishes*.
Herbs, spices, condiments	Herbs, spices, condiments	Seasoning, dried spices and condiments, e.g., cinnamon, oregano (dried), thyme (dried), taco spice mix, vegetable stock, apple cider vinegar, rice vinegar, soya sauce, salt.
Composite meals	Composite meals*	Composite meals which could not be disaggregated into ingredients, i.e., gyoza dumplings, spring rolls, miso soup, quorn lasagne (frozen), vegetable and soy mince pirogi, savoury cheese pie.

*Includes vegan alternatives. †Whole grain products are defined as having >50% (dry weight) whole grains, which was determined by using the Swedish Food Composition database as well as brand product information. ‡Pseudocereals refers to foods which are consumed like grains and cereals, and are classified as whole grains, but botanically differ from grains. ¶Includes processed products of poultry/meat. §Not including dairy ice-cream or puddings (ice-cream and pudding group) or butter (butter and margarine group).

Supplementary Table 3. Absolute (g/d) usual daily intake of food groups (mean±SD) among youth aged 16 to 24 years in Sweden adhering to vegan, lacto-ovo-vegetarian, pescatarian or omnivorous diets (n=235).

Food group	Total sample (n=235)		Vegan (n=60)		Lacto-ovo-vegetarian (n=59)		Pescatarian (n=55)		Omnivore (n=61)		
Absolute usual daily intake, g/d	**Mean**	**SD**	**Mean**	**SD**	**Mean**	**SD**	**Mean**	**SD**	**Mean**	**SD**	***P*-value***
**Plant-based foods**											
Vegetables, root vegetables, and mushrooms (all types)[†]	213	83	227	82	213	89	202	73	210	86	0.42
Fruits and berries[††]	121	95	139	127	113	68	115	91	117	85	0.41
Fruit and vegetable juice	33	56	42	80	40	47	23	35	28	50	0.19
Potatoes, plain	31	30	28	24	42[a]	36	31	23	23[b]	32	**0.006**
Fried potatoes and potato dishes[‡]	26	27	19[a]	22	19[a]	30	31	28	35[b]	23	**<0.001**
Whole grain products[§]	57	59	47	39	44[a]	35	75[b]	94	63	48	**0.015**
Refined grain products	203	79	231[a]	90	202	85	179[b]	54	196	73	**0.004**
Legumes	43	37	88[a]	38	41[b]	27	33[b]	9	11[c]	6	**<0.001**
Nuts and seeds[¶]	15	18	20	22	13	12	13	18	12	17	0.07
Vegetable and seed oils	9	7	11[a]	9	10	7	9	6	7[b]	7	**0.023**
Plant-based meat analogs	63	56	118[a]	55	78[b]	44	44[c]	29	9[d]	20	**<0.001**
Plant-based dairy substitutes	115	116	221[a]	130	124[b]	100	65[c]	72	47[c]	61	**<0.001**
**Animal-sourced foods**											
Red meat and poultry (all types, including processed)	39	70	0[a]	0	0[a]	0	0[a]	0	149[b]	49	**<0.001**
Fish, seafood, and fish products (all types)	15	27	0[a]	0	0[a]	0	35[b]	36	25[b]	28	**<0.001**
Egg (all types)	17	22	0[a]	0	19[b]	27	23[b]	20	25[b]	22	**<0.001**
Milk and dairy products[‖]	172	179	0[a]	0	175[b]	106	228[b,c]	120	289[c]	235	**<0.001**
Butter and margarine[‡]	10	6	9[a]	6	9[a]	6	11[a]	8	9[a]	5	0.07
**Sweets and snack foods and beverages**											
Candy and chocolate products[‡]	18	17	10[a]	10	17[a,b]	11	21[b,c]	15	26[c]	23	**<0.001**
Cakes, baked goods, sweet snack bars[‡]	34	23	40[a]	17	27[b]	28	36	22	31	22	**0.019**
Ice-cream and cream-based puddings[‡]	13	20	13	27	14	12	12	18	12	21	0.97
Salted snacks[‡]	14	13	14	9	15	14	10	8	15	16	0.11
Sugar-sweetened beverages	64	65	72	82	66	75	70	43	50	48	0.23
Alcoholic beverages	96	104	78[a]	81	139[b]	130	105	115	66[a]	67	**<0.001**

g/d, gram per day. SD, Standard deviation.

Usual daily intakes were calculated by the Multiple Source Method using repeated 24-h dietary recalls (between 2-4) and consumption frequency of food groups from a dietary screener. For the food groups, refined grains, vegetable and seed oils, and eggs, consumption frequency was not assessed. [*]For all variables, to test for differences between dietary groups one-way ANOVA was used with Bonferroni *post hoc* test to adjust for multiple comparisons. Bolded p-values indicate significant difference between the groups in the *post hoc* test and unlike letters ([a,b,c,d]) in the same row indicate between which dietary groups there is a difference. [†]Includes processed vegetable products and excludes potatoes and legumes. [††] Includes fresh, frozen, canned, and dried fruit/berries, and excludes fruit juices (separate group) and fruit/berry jams, marmalades, and compotes (separate group). [‡]Includes vegan alternatives. [§]Whole grain products are defined as having >50% (dry weight) whole grains (32). [¶]Includes salted nuts/seeds. [‖]Includes cheese and cheese products and excludes dairy ice-cream (ice-cream and cream-based pudding group) and butter (butter and margarine group). For details of foods included in each food group, refer to supplementary material **Table 2**. *P*-value of <0.05 (two-sided) was accepted as statistically significant.

Supplementary Table 4. Energy-density (g/MJ) of usual daily intakes of food groups (mean±SD) among youth aged 16 to 24 years in Sweden adhering to vegan, lacto-ovo-vegetarian, pescatarian or omnivorous diets (n=235).

Food group	Total sample (n=235)		Vegan (n=60)		Lacto-ovo-vegetarian (n=59)		Pescatarian (n=55)		Omnivore (n=61)		
Energy-density of usual daily intake, g/MJ	**Mean**	**SD**	**Mean**	**SD**	**Mean**	**SD**	**Mean**	**SD**	**Mean**	**SD**	***P*-value***
**Plant-based foods**											
Vegetables, root vegetables, and mushrooms (all types)[†]	23	9	24	8	24	10	23	9	21	8	0.32
Fruits and berries[††]	13	10	15	13	13	8	13	10	12	9	0.43
Fruit and vegetable juice	3	6	4	7	4	6	2	4	3	5	0.09
Potatoes, plain	3	3	3	3	4[a]	4	3	2	3[b]	3	**0.002**
Fried potatoes and potato dishes[‡]	3	3	2	3	2	3	4	3	4	2	0.007
Whole grain products[§]	6	7	5[a]	4	5[a]	4	9[b]	11	6	4	**0.011**
Refined grain products	21	7	24[a]	8	22[a,b]	7	20[b]	5	20[b,c]	7	**0.001**
Legumes	5	4	9[a]	4	5[b]	3	4[b]	1	1[c]	1	**<0.001**
Nuts and seeds[¶]	2	2	2[a]	2	1	1	1	2	1[b]	2	**0.033**
Vegetable and seed oils	1	1	1[a]	1	1	1	1	1	1[b]	1	**0.006**
Plant-based meat analogs	7	6	12[a]	5	9[b]	5	5[c]	4	1[d]	2	**<0.001**
Plant-based dairy substitutes	13	13	24[a]	13	14[b]	12	7[c]	8	5[c]	6	**<0.001**
**Animal-sourced foods**											
Red meat and poultry (all types, including processed)	4	7	0[a]	0	0[a]	0	0[a]	0	15[b]	5	**<0.001**
Fish, seafood, and fish products (all types)	2	3	0[a]	0	0[a]	0	4[b]	4	3[c]	3	**<0.001**
Egg (all types)	2	2	0[a]	0	2[b]	3	3[b]	2	3[b]	2	**<0.001**
Milk and dairy products[‖]	18	17	0[a]	0	19[b]	10	25[b,c]	13	28[c]	21	**<0.001**
Butter and margarine[‡]	1	1	1[a]	1	1[a,b]	1	1[b]	1	1[a]	0	**0.009**
**Sweets and snack foods and beverages**											
Candy and chocolate products[‡]	2	2	1[a]	1	2[a,b]	1	2[b]	2	3[b,c]	2	**<0.001**
Cakes, baked goods, sweet snack bars[‡]	4	2	4	2	3	3	4	2	3	2	0.02
Ice-cream and cream-based puddings[‡]	1	2	1	3	2	1	1	2	1	2	0.83
Salted snacks[‡]	1	1	1	1	2	2	1	1	2	1	0.24
Sugar-sweetened beverages	7	7	8	9	7	9	8	5	5	5	0.13
Alcoholic beverages	11	12	8[a]	9	16[b]	16	12	11	7[a]	7	**<0.001**

g/MJ, Gram per Megajoule. SD, Standard deviation.

Usual daily intakes were calculated by the Multiple Source Method using repeated 24-h dietary recalls (between 2-4) and consumption frequency of food groups from a dietary screener. For the food groups, refined grains, vegetable and seed oils, and eggs, consumption frequency was not assessed. [*]For all variables, to test for differences between dietary groups one-way ANOVA was used with Bonferroni *post hoc* test to adjust for multiple comparisons. Bolded p-values indicate significant difference between the groups in the *post hoc* test and unlike letters ([a,b,c,d]) in the same row indicate between which dietary groups there is a difference. [†] Includes processed vegetable products and excludes potatoes and legumes. [††] Includes fresh, frozen, canned, and dried fruit/berries, and excludes fruit juices (separate group) and fruit/berry jams, marmalades, and compotes. [‡]Includes vegan alternatives. [§]Whole grain products are defined as having >50% (dry weight) whole grains (32). [¶]Includes salted nuts/seeds. [‖]Includes cheese and cheese products and excludes dairy ice-cream (ice-cream and cream-based pudding group) and butter (butter and margarine group). For details of foods included in each food group, refer to supplementary material **Table 2**. *P*-value of <0.05 (two-sided) was accepted as statistically significant.

Supplementary Table 5. Usual daily intakes of energy, macronutrients, whole grain and salt (mean±SD) among youth aged 16 to 24 years in Sweden adhering to vegan, lacto-ovo-vegetarian, pescatarian or omnivorous diets (n=235).

		Total sample (n=235)		Vegan (n=60)		Lacto-ovo-vegetarian (n=59)		Pescatarian (n=55)		Omnivore (n=61)		
**Usual daily intake**	**Recommended daily intake[*]**	**Mean**	**SD**	**Mean**	**SD**	**Mean**	**SD**	**Mean**	**SD**	**Mean**	**SD**	***P*-value[†]**
Energy, MJ/d[‡]	9.4 MJ (Female), 11.8 MJ (Male)[*]	10	2	10	2	9	2	9	2	10	2	0.048
Protein, g/d		79	27	70[a]	23	73[a]	23	76[a]	19	98[b]	31	**<0.001**
Protein, E%	10-20 E%	14	3	12[a]	3	13[a,b]	3	14[b]	3	16[c]	3	**<0.001**
Protein, g/kg[‡]	≥0.83 g/kg[§]	1.2	0.4	1.1[a]	0.3	1.2[a]	0.4	1.2[a]	0.3	1.4[b]	0.4	**<0.001**
Carbohydrates, g/d		246	58	270[a]	64	239[b]	52	228[b]	40	247	65	**<0.001**
Carbohydrates, E%	45-60 E%	46	7	51[a]	6	46[b]	6	44[b,c]	6	43[c]	6	**<0.001**
Dietary fiber, g/d	≥25 g/d (Female), ≥35 g/d (Male)	29	9	36[a]	9	29[b]	8	26[b]	9	26[b]	8	**<0.001**
Dietary fiber, g/MJ[‡]	≥3 g/MJ	3	0.8	3.8[a]	1	3.2[b]	1	2.9[b]	0.8	2.5[c]	1	**<0.001**
Total sugars, g/d		81	22	82	27	78	14	78	16	84	28	0.30
Total sugars, E%		14	3	15	4	14	3	15	3	14	3	0.73
Free sugars, g/d[ǀ]		39	14	39	14	39	8	38	11	40	20	0.92
Free sugars, E%[ǀ]	<10 E%	7	2	7	3	7	2	7	2	7	3	0.54
Fat, total, g/d		96	26	91[a]	23	94	30	95	26	104[b]	22	**0.024**
Fat, total, E%	25-40 E%	38	5	36[a]	5	38	5	39[b]	5	39[b]	5	**<0.001**
SFA, g/d		31	11	22[a]	7	30[b]	12	33[b]	9	38[c]	10	**<0.001**
SFA, E%	<10 E%	12	3	9[a]	2	12[b]	3	13[b,c]	3	14[c]	3	**<0.001**
MUFA, g/d		40	11	40	11	39	12	39	12	43	9	0.17
MUFA, E%	10-20 E%	16	3	16	3	16	2	16	2	16	2	0.84
PUFA, g/d		19	6	23[a]	6	18[b]	6	17[b]	5	16[b]	4	**<0.001**
PUFA, E%	5-10 E%	7	2	9[a]	2	7[b]	2	7[b]	1	6[c]	1	**<0.001**
Whole grain, g/d[¶]	≥90 g/d	50	33	47	23	44	29	49	36	58	40	0.13
Salt, g/d[††]	<6 g/d	8	2	8	2	7	2	7[a]	2	8[b]	2	**<0.001**

MJ/d, Megajoule per day. SD, Standard deviation. SFA, Saturated fatty acids. MUFA, Monounsaturated fatty acids. PUFA, Polyunsaturated fatty acids.

Usual daily intakes were calculated using the Multiple Source Method based on repeated 24-h dietary recalls (between 2-4 days). [*]Recommended dietary intake by NNR2023 for healthy 18–24-year-olds, and recommended energy intake is based on a standard weight and physical activity level of 1.6. [†]For all variables, to test for differences between diet groups one-way ANOVA was used with Bonferroni *post hoc* test to adjust for multiple comparisons. Bolded p-values indicate significant difference between the groups in the *post hoc* test, and unlike letters ([a,b,c]) in the same row indicate between which dietary groups there is a difference. [‡]Values are given with one decimal place for meaningful values. [§]Recommended daily intake of protein in gram per kilogram bodyweight for adults ≥18 years, both sexes. [ǀ]Free sugars was defined according to WHO´s definition (31), i.e., sugars from all foods which contain added sugars, as well as sugars which are naturally present in honey, syrups, fruit juice and fruit juice concentrate. [¶]Content of whole grains in foods was automatically calculated using the Swedish Food Composition database or by brand product information. [††]Salt from foods only (including salt in the cooking method, e.g., ´cooked pasta with salt´), and not including discretionary sources of salt reported in the 24HDR´s. *P*-value of <0.05 (two-sided) was accepted as statistically significant.
